# Continuous Liquid–Liquid Extraction of High-Purity Lutein from *Chlorella vulgaris* via Centrifugal Partition Chromatography: Utilizing Limonene as Renewable Solvent for Microalgae Biomass Valorization

**DOI:** 10.3390/foods14091637

**Published:** 2025-05-06

**Authors:** Weiheng Kong, Xianjiang Lin, Jing Ye, Yanbin Lu

**Affiliations:** 1 Science and Technology Research Center of China Customs, Beijing 100026, China; 2 National R&D Branch Center for Marine Fish Processing, Zhejiang Provincial Key Laboratory of Food Microbiology and Nutritional Health, Institute of Seafood, Zhejiang Gongshang University, Hangzhou 310018, China; 24020080027@pop.zjgsu.edu.cn (X.L.); yellowleaf@zjgsu.edu.cn (J.Y.)

**Keywords:** eco-friendly, citrus waste, limonene-based biphasic system, solid support-free technique, sustainable separation

## Abstract

In this study, an efficient and eco-friendly method was developed for continuous liquid–liquid extraction of lutein from microalgae *Chlorella vulgaris*. By employing a limonene-based biphasic liquid system, high-purity lutein was successfully obtained from the crude extract in a single run via centrifugal partition chromatography (CPC). Evaluation and optimization results demonstrated that limonene could effectively serve as a replacement for *n*-hexane as the solvent system for lutein extraction, exhibiting natural renewability and minimal environmental impact. Furthermore, the elution–extrusion operation mode was employed to fully exploit the liquid nature of the stationary phase in the extraction process, allowing for continuous sampling and separation without interruption. This proposed protocol offers a sustainable and environmentally friendly alternative for extracting valuable ingredients from microalgae biomass, demonstrating its potential as a scalable solution for producing lutein-enriched ingredients applicable to functional foods and nutraceuticals.

## 1. Introduction

Microalgae, such as *Chlorella vulgaris*, are unicellular photosynthetic organisms that have gained increasing attention due to their high content of bioactive compounds [1]. *C. vulgaris* is recognized for its diverse array of bioactive molecules, including proteins, carbohydrates, lipids, carotenoids, and pigments, as well as vitamins and minerals [2]. These compounds demonstrate a broad spectrum of biological activities, including antioxidant, anti-inflammatory, anticancer, and immunomodulatory effects [3,4]. Lutein, as the main xanthophyll carotenoid, is present in high concentrations in *C. vulgaris* [5]. By collecting light energy and shielding the photosynthetic machinery from photo-oxidative damage, this yellow pigment is essential to the process of photosynthesis [6]. Apart from its role in photosynthesis, lutein has been found to have various health benefits. It is a potent antioxidant that can scavenge free radicals and safeguard cells from oxidative stress [7,8]. Lutein has also been shown to have anti-inflammatory properties and can modulate immune function [9,10]. Moreover, lutein is recognized for its protective benefits on eye health, especially in preventing age-related macular degeneration and cataracts [11,12]. Lutein is believed to possess potential benefits in preventing and treating several diseases, including cardiovascular disease, diabetes, and cancer [13,14]. Consequently, lutein is recognized as a valuable bioactive compound with promising therapeutic applications. It has extensive applications across the food industry, dietary supplements, and pharmaceutical fields.

At present, the major sources of lutein are marigold flowers (*Tagetes erecta*) and green leafy vegetables, such as kale and spinach [15]. Marigold flowers serve as the main source of lutein for supplements and functional foods, while green leafy vegetables provide the majority of lutein in human diets [16]. In recent years, microalgae, such as *C. vulgaris*, have emerged as a potential source of lutein due to their high lutein content and rapid growth rate [17]. Microalgae-derived lutein has several advantages over traditional sources, including year-round availability, consistent quality, and lower environmental impact [14]. Moreover, microalgae-derived lutein has been shown to have higher bioavailability and efficacy than that from other sources [18]. The growing need for natural and functional ingredients in the food and dietary supplement industries is expected to propel the worldwide lutein market’s growth in the next years. With the rapid increase in demand for lutein in the market, the efficient extraction and production of lutein from microalgae have become increasingly important.

Centrifugal partition chromatography (CPC) functions as a continuous and efficient liquid–liquid partition chromatographic separation technique, akin to the hydrostatic countercurrent chromatography (CCC) column, and it does not require solid carriers or support [19]. The centrifuge rotor used in CPC is composed of a series of disks that are engraved with interconnected chambers and ducts. The design of these chambers ensures the stationary phase remains under a constant centrifugal force. CPC can be considered as enhanced Craig machines, featuring linked test tubes capable of functioning at increased mobile phase flow rates because of the much stronger centrifugal field produced by the CPC [20]. In terms of strength, this field greatly exceeds the gravitational field employed in the Craig machine. Compared to traditional liquid–solid chromatography, CPC offers several unique advantages [21]. It does not rely on solid supports in the pipeline, instead separating substances based on their partition coefficients (*K_D_*) in the two phases [22]. This approach avoids irreversible adsorption-induced sample denaturation, loss, and denaturation problems. CPC also uses a two-phase solvent system, which increases the polarity range and expands its applicability. Additionally, due to the full contact between the target and the liquid stationary phase, CPC allows for larger sample loading volumes. The operating cost of CPC is relatively low, and the sample pretreatment process is simple, with flexible and diverse elution methods. Given these benefits, CPC is increasingly utilized for isolating active components from natural products, highlighting its potential for future industrial applications [23,24].

**Figure 1:**
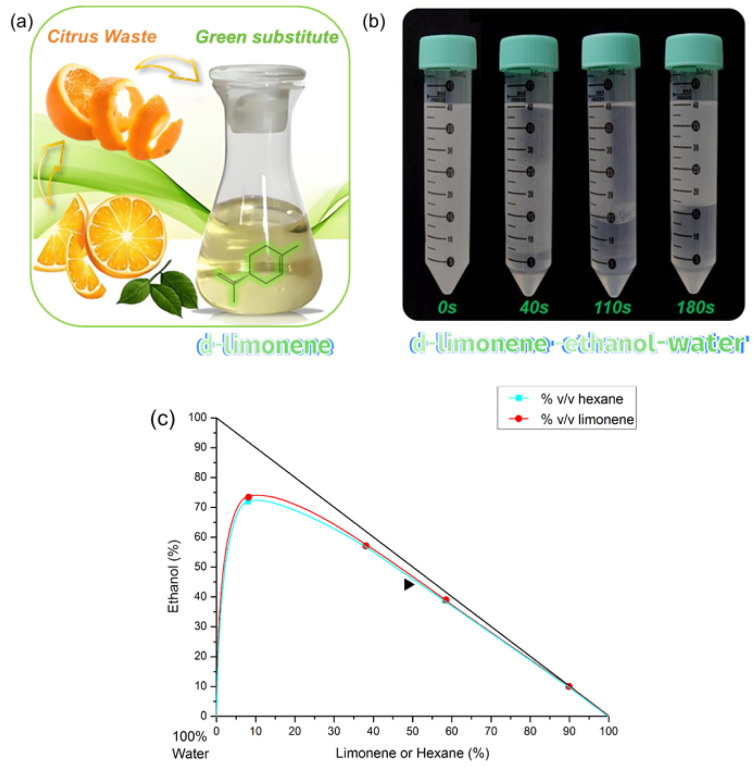
(**a**) The main resource and chemical structure of limonene; (**b**) the liquid–liquid equilibrium of limonene–ethanol–water (10:9:1, *v*/*v*) biphasic liquid system; (**c**) the ternary phase diagram of limonene/hexane–ethanol–water solvent system.

However, a potential drawback of CPC is its reliance on large volumes of organic solvents, which can be a concern for both economic and environmental reasons. CPC typically uses a biphasic solvent system consisting of two immiscible liquid phases, such as *n*-hexane, methanol, and ethyl acetate [21,22]. Large amounts of the mobile phase are generally used to elute the target compounds from the column. This can result in a significant consumption of organic solvents, which can be expensive and have environmental impacts. To make the CPC separation process more environmentally friendly, it is essential to seek renewable, natural reagents to replace organic reagents [25]. Limonene (shown in Figure 1a), a pale yellow liquid characterized by its pleasant aroma, is an exemplary candidate owing to its natural abundance, superior quality, and inherent safety [26]. This substance has a molecular weight of 136.24 g/mol and a molecular formula of C_10_H_16_. It is widely found in nature, particularly in citrus waste (peel crude oil), with a content of up to 90%, and mainly exists in the structure of the *d*-limonene isomer. Limonene has several unique physicochemical properties that make it a potential green, biodegradable, and renewable solvent that can be easily recovered and recycled for various industrial applications [27]. It serves as a solvent for degreasing and cleaning across various industries, such as electronics, automotive, and aerospace. It can also be used as a solvent for extraction and purification in the food, pharmaceutical, and cosmetic industries. Faure et al. reported the possibility of using limonene instead of alkane in CCC and systematically studied the “Arizona” quaternary solvent systems based on limonene [28,29,30]. However, at present, there are rarely reported CCC/CPC separation cases using limonene-based two-phase solvent systems in real samples. In this study, a fully environmentally friendly liquid–liquid extraction protocol via CPC for separation of high-purity lutein from *C. vulgaris* biomass was proposed. This was achieved by comparing the phase diagrams of the limonene–ethanol–water and *n*-hexane–ethanol–water solvent systems, systematically analyzing the physical/chemical properties of limonene and *n*-hexane, determining the partition coefficient of lutein, and optimizing various parameters and conditions of CPC to determine the best separation conditions. Given its numerous advantages, it is anticipated that this approach will provide a sustainable and efficient alternative for extracting and purifying value-added ingredients from microalgae biomass.

## 2. Materials and Methods

### 2.1. Materials and Reagents

The biomass of *C. vulgaris* was provided by Xindaze Spirulina Co., Ltd. (Fuqing, China). The carotenoid content was approximately 4.5 mg/g dried biomass, as reported by the producer. The sample was stored at 4 °C under dark conditions for future use. Lutein (all-trans, 90% purity) was sourced from Tauto Biotech Co., Ltd. in Shanghai, China. HPLC-grade acetonitrile and methanol were purchased from Merck, Darmstadt, Germany. Ultrapure water with a resistivity of 18.2 MΩ cm^−1^ was generated using a Milli-Q water system. d-limonene was purchased from Aladdin Biochemical Technology (Shanghai, China) at 95% purity. Other solvents for sample preparation and CPC separation were all obtained from Huadong Chemicals (Hangzhou, China).

### 2.2. Sample Preparation

According to our previous report [31], *C. vulgaris* biomass was gathered, freeze-dried using a vacuum freeze-dryer, and kept in the dark under vacuum conditions. A 50.0 g sample of the freeze-dried substance was extracted using a 0.5 L *n*-hexane/ethanol (1:1) solution for 2 h at 35 KHz with an ultrasonic device (KQ500E Ultrasonics, Kunshan, China). After three extraction rounds, the combined extracts were merged and evaporated to dryness at 35 °C using a rotary evaporator, yielding 6.08 g of crude *C. vulgaris* extract, which was then stored at −20 °C until use.

### 2.3. HPLC Analysis

A Waters Acquity ultra-high pressure LC system was used to analyze the crude *C. vulgaris* extract and the purified CPC fractions as follows [31]: The separation was carried out using a T3 column (HSS type, 2.1 × 100 mm i.d., 1.8 μm, Waters) with an on-line filter. The mobile phase was composed of A (methanol/acetonitrile, 3:7) and B (water) with this gradient: 0 to 5 min, 50% A; 5 to 10 min, 50 to 80% A; 10 to 35 min, 80 to 100% A; 35 to 40 min, 100% A; 40 to 43 min, 100 to 50% A; 43 to 50 min, 50% A. The column was maintained at 35 °C, injection volume: 10 μL, and the effluent was monitored at 450 nm with a flow rate of 0.3 mL per minute. The lutein content was simply determined by HPLC peak area using the standard as a reference.

### 2.4. Limonene-Based Green Biphasic Liquid Systems

#### 2.4.1. Ternary Phase Diagram

The phase diagram is an intuitive and effective method for describing the composition of biphasic solvent systems. On the other hand, the ternary phase diagram describes the composition of ternary solvent systems consisting of three solvent components A, B, and C. The relationship between them can be expressed as follows:

A% + B% + C% = 100%(1)

The percentages can be expressed in terms of mass, molecular weight, or volume. The ternary diagram is a map that shows the range of monophasic, biphasic, and multiphase compositions of the three components in a system. This study utilized volume percentages to construct ternary phase diagrams for the two studied biphasic liquid systems composed of *n*-hexane–ethanol–water and limonene–ethanol–water. Among the substances involved, A, B, and C represent water, ethanol, and limonene/*n*-hexane, respectively. Four mixed solvents of B-C with volume ratios of 9:1, 6:4, 4:6, and 1:9 (*v*/*v*) were prepared in separate test tubes, and a small amount of known volume of A was added to each tube. The tube was shaken vigorously and observed after standing. If there was no phase separation (turbid state), A was added until phase separation occurred. At this point, the volume of A added was known, as well as the volumes of B and C, and the tie line (the border between single-phase and two-phase sections) was plotted to create the ternary phase diagram. The experimental temperature was maintained at approximately 25 ± 2 °C.

#### 2.4.2. Phase Composition Determination

The phase compositions of biphasic liquid systems, specifically *n*-hexane–ethanol–water (10:9:1) and limonene–ethanol–water (10:9:1), were systematically examined. The solvent mixtures were equilibrated at room temperature in a 25 mL vial for two hours prior to phase composition analysis. The contents of the vial were poured into a 25 mL graduated cylinder to allow phase separation, and the relative volumes of the phases were measured directly. The density of each phase was found by weighing 10 mL of each in a calibrated vial. The water content in both the upper and lower phases was determined using a Karl Fischer moisture analyzer from METTLER TOLEDO, Shanghai, China. Organic compounds like ethanol and limonene were detected using an Agilent 7890A gas chromatograph equipped with an FID detector. The analytical setup comprises an HP-INNOWAX capillary column (30 m × 0.25 mm, 0.25 μm) with an injection port temperature of 250 °C; an injection volume of 1 μL; a split ratio of 200:1; and high-purity nitrogen serving as the carrier gas. The oven was initially set to 50 °C, maintained for 1 min, then increased by 10 °C per minute until reaching 150 °C in 10 min, held at 150 °C for 2 min, and finally returned to 50 °C for the subsequent cycle.

### 2.5. Determination of Partition Coefficient (K_D_)

The partition coefficient (*K_D_*) is a solute physicochemical parameter that determines its retention in CCC, making it a crucial index for evaluating the suitability of a solvent system. The *K_D_* value of lutein was determined according to the literature as follows [32]: 1.0 mL of each phase was utilized to dissolve 1.0 mg of *C. vulgaris* extract. The mixture was separated using a centrifuge at 3000 rpm for 5 min. The *K_D_* value was calculated using HPLC by comparing the peak area of the target compound in the organic phase to that in the aqueous phase.

### 2.6. HPCPC Separation

#### 2.6.1. Instrument

Lutein was continuously extracted from *C. vulgaris* biomass using a High-Performance CPC instrument (HPCPC-240, Sanki Engineering, Kyoto, Japan). The rotor of the CPC-240 comprises 12 disks, each containing 178 partition cells, resulting in a total column volume of 240 mL (*V_C_* = 240 mL). The CPC device includes a four-way switching valve that allows it to function in both descending and ascending modes. With a speed controller, the apparatus’s revolution speed can be set anywhere from 0 to 2000 rpm. The system includes two Model P230 pumps and a Model UV 200 II ultraviolet detector from Elite, Dalian, China. A 10 mL sample loop injection valve was utilized for sample introduction. The effluent was continuously collected using a Waters fraction collector III, while the elution profile was recorded with an HS 2000 data analysis system.

#### 2.6.2. Preparation of Biphasic Solvent System and Sample Solution

The biphasic liquid system used for CPC separation was mixed according to the selected volume ratio. After thorough mixing at room temperature, the newly prepared biphasic solvent system was allowed to stand and layer. The solvents from the upper and lower phases were then separated for later use. A specific quantity of the substance was dissolved in a solvent made of equal parts of the upper and lower phases. The sample solution was then prepared and stored for later use.

#### 2.6.3. CPC Procedures

Initially, the CPC column was loaded with the selected stationary phase. Subsequently, the rotor was adjusted to the correct speed, and the mobile phase was pumped in the designated direction at the chosen flow rate. Using the upper mobile phase, the ascending flow direction was chosen in normal phase mode. A graduated cylinder measured the volume of stationary phase displaced by the mobile phase at the column outlet. When a meniscus emerged in the cylinder, it signified that hydrodynamic equilibrium was achieved, with the mobile phase leaving the column outlet instead of moving the stationary phase. In the equilibrated column, the volume of the mobile phase, *V_M_*, corresponded to the collected volume of the displaced stationary phase. After achieving hydrodynamic equilibrium, the sample solution was introduced into the column. The effluent was collected using a fraction collector and continuously observed at a wavelength of 450 nm.

#### 2.6.4. Elution–Extrusion Separation Mode

Considering the complexity of the crude *C. vulgaris* extract, this study employed the CPC separation process in an elution–extrusion mode to expand its hydrophobicity range, as documented in previous studies [32,33]. Initially, the separation process was originally developed in classical mode, utilizing a fixed volume of the mobile phase, referred to as CM. The next step involved injecting the stationary phase into the column rather than the mobile phase, maintaining the same flow rate, flow direction, and rotor rotation speed. The extrusion step began at this point, with the stationary phase retaining all remaining solutes in the column after the mobile phase was flushed out during an intermediate step. The CPC column was prepared for subsequent sample injections following the elution of each solute with approximately one column volume of stationary phase, thereby ensuring a continuous separation process.

## 3. Results and Discussion

### 3.1. Physicochemical Properties of Limonene-Based Green Biphasic Liquid Systems

Limonene is a nonpolar solvent that is highly miscible with nonpolar compounds, such as oils, waxes, and resins. Table 1 presents a comparison of the physicochemical properties of d-limonene with *n*-hexane, ethanol, and water. Additionally, it compares the phase composition in both of the limonene–ethanol–water and *n*-hexane–ethanol–water systems with the same volume ratio (10:9:1, *v*/*v*). Table 1 indicates that limonene and *n*-hexane exhibit comparable physical and chemical properties, with analogous parameters observed in the two biphasic liquid systems under study. The main difference between limonene and *n*-hexane is their density, with limonene having a density 24% higher than *n*-hexane. In the limonene–ethanol–water (10:9:1, *v*/*v*) system, the lower phase is organic and rich in limonene, whereas in the *n*-hexane–ethanol–water (10:9:1, *v*/*v*) system, the upper phase is organic and rich in *n*-hexane. In CPC, the flow direction of the mobile phase is primarily influenced by the density difference between the stationary and mobile phases. If the lower phase is the mobile phase, a reverse-phase mode must be used for elution, and vice versa [34,35,36]. In the *n*-hexane–ethanol–water (10:9:1, *v*/*v*) system, utilizing *n*-hexane as the stationary phase necessitates a reverse-phase elution mode. In the limonene–ethanol–water (10:9:1, *v*/*v*) system, selecting the limonene-rich lower phase as the stationary phase necessitates using a normal-phase elution mode.

According to Table 1, the density difference between the upper and lower phases in the limonene–ethanol–water (10:9:1, *v*/*v*) mixture is minimal (0.009 g/cm^3^), leading to the creation of distinctive foam in the lower limonene phase (Figure 1b). Traditional hydrodynamic CCC columns require a significant density difference between the upper and lower phases to achieve a high retention rate of the stationary phase. The higher density of limonene compared to *n*-hexane poses a challenge for hydrodynamic CCC columns, which need a minimum phase-density difference to maintain an adequate liquid stationary phase. In contrast, the minimal density difference in the limonene–ethanol–water (10:9:1, *v*/*v*) biphasic system benefits hydrostatic CPC columns, as it enhances the driving pressure of the mobile phase. By utilizing maximum centrifugal fields, substantial amounts of retained stationary phase can be achieved with hydrostatic CPC apparatus [28].

### 3.2. Comparison of the Ternary Phase Diagram

The ternary phase diagram describes the composition of a ternary solvent system, which is composed of three kinds of solvents. Figure 1c illustrates the ternary phase diagrams of the limonene–ethanol–water and *n*-hexane–ethanol–water solvent systems, both of which are classified as orthogonal type. The origin of coordinates represents a water content of 100%, and the diagonal lines far from the origin and connecting the two rectangular coordinate systems represent a water content of 0%. The diagram indicates that the limonene–ethanol–water system predominantly exhibits a two-phase system in most areas, suggesting that it will demonstrate a layering phenomenon under various volume ratios. The limonene–ethanol–water solvent system is very similar to the *n*-hexane–ethanol–water solvent system, with the only difference being that the solubility of ethanol in limonene is greater than in *n*-hexane. The solid triangle in the figure represents the limonene–ethanol–water system (10:9:1, *v*/*v*) system capable of forming a biphasic system. Thus, it can be inferred that limonene has the potential to replace *n*-hexane as the solvent system for CPC separation. However, the ternary solvent system is not optimal for CPC separation due to the significant difference in polarity between the two-phase solvents. The commonly used “Arizona” system or “HEMWat” system are both quaternary solvent systems, widely employed for separating samples with varying polarities due to their broad polarity range [22]. Therefore, it is essential for future research to investigate and utilize the limonene-based quaternary solvent system.

### 3.3. Partition Coefficient of Lutein in Limonene-Based Liquid System

Effective CPC separation requires a thorough grasp of liquid–liquid equilibrium and solute partitioning theory when using a biphasic liquid system. Any modification in the composition of the mobile phase has the potential to influence the composition of the stationary phase, which may result in a loss of hydrodynamic equilibrium and overall separation efficiency. Consequently, the partition coefficient (K_D_) of the target analyte is a critical parameter that is exclusively dependent on the chosen biphasic liquid systems. The wide variety of potential solvent systems can complicate CPC method development. It has been estimated that approximately 90% of all efforts in CPC are devoted to solvent selection. For effective separation, the K_D_ value for the target compound should ideally be between 0.5 and 2.0. For satisfactory peak resolution, the selectivity factor (α = K_D2_/K_D1_) between any two components should be greater than 1.5. In this study, the extremely low polarity of lutein prompted us to initially consider a hydrophobic ternary solvent system. Furthermore, we assessed the potential use of environmentally friendly limonene as a substitute for *n*-hexane. The partition coefficient of lutein is presented in Table 2. It is evident from the table that due to limonene’s higher polarity compared to *n*-hexane, the K_D_ value of lutein in the limonene–ethanol–water solvent system surpasses that in the *n*-hexane–ethanol–water solvent system with an equivalent volume ratio. The partition coefficient of lutein in the limonene–ethanol–water (15:9:1, *v*/*v*) system is notably small and unsuitable for separating lutein. Increasing the water phase proportion optimizes the partition coefficient of lutein in the limonene–ethanol–water (10:9:1, *v*/*v*) system, enhancing its suitability for separation. Using a limonene–ethanol–water (10:7:3, *v*/*v*) system results in a high partition coefficient, complicating lutein elution and causing broader chromatographic peaks. Consequently, the experimental results led to the selection of the limonene–ethanol–water (10:9:1, *v*/*v*) system as the optimal solvent for lutein separation.

### 3.4. Stationary Phase Retention of Limonene-Based Liquid System

Retention of the stationary phase (RSP) is crucial for enhancing the separation efficiency in CPC. The retention factor (S_f_) is calculated using the following equation:

*S*_f_ = V_S_/V_C_ = 1 − V_M_/V_C_ (2)

where V_C_: column volume; V_S_: volume of stationary phase in the chromatographic column; V_M_: volume of mobile phase in the chromatographic column.

The retention of the stationary phase within the column plays a crucial role in influencing peak resolution, with increased retention levels correlating to enhanced peak resolution. Generally, an RSP exceeding 40% yields improved separation effects, whereas an RSP below 40% results in a loss of stationary phase and significantly diminishes the separation efficacy [37]. Previous research has demonstrated that the RSP is influenced by variations in both the revolution speed and flow rate of the mobile phase [38]. Consequently, these two parameters were optimized in this study.

#### 3.4.1. Effect of Rotation Speed

**Figure 2:**
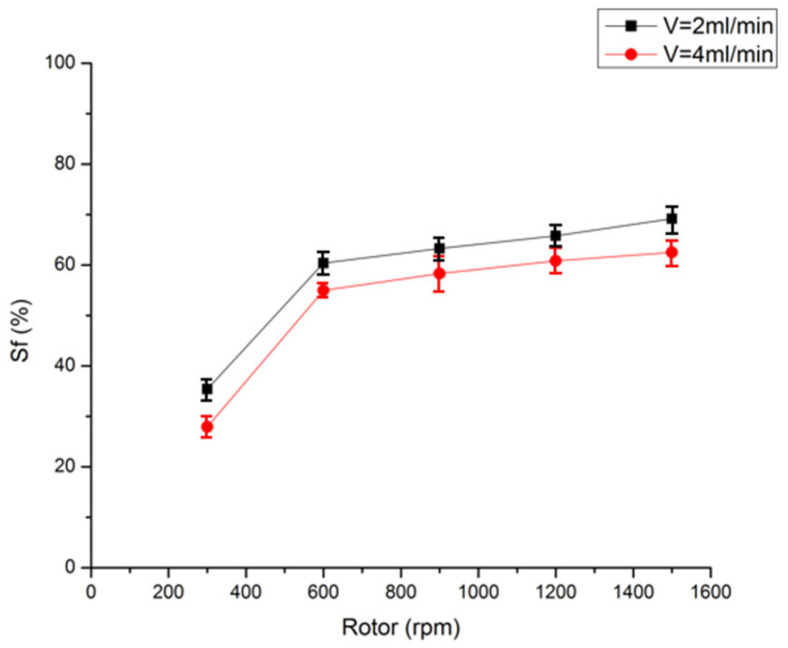
Effects of rotational speed on the retention of stationary phase (flow rate: 2.0 mL/min and 4.0 mL/min).

Figure 2 illustrates the impact of CPC rotation speed on the stationary phase retention rate at mobile phase flow rates of 2.0 mL/min and 4.0 mL/min with a 50 mg injection. The figure shows that the stationary phase retention rate is notably higher at a flow rate of 2.0 mL/min than at 4.0 mL/min, given the same rotation speeds. At a flow rate of 2.0 mL/min, increasing the rotation speed consistently improves retention rates. For instance, when increasing from 300 rpm to 600 rpm, there is a notable rise from 35.4% to 60.4%. However, for rotation speeds equal to or exceeding 600 rpm, only marginal increases in retention rates are observed with further increments in rotation speed; for example, transitioning from 600 rpm to 1500 rpm results in an increase from 60.4% to just 69.3%. While it is clear that elevating rotation speed can improve retention rates for the stationary phase, it is crucial to acknowledge that system pressure will also escalate with increased rotational velocities; excessive pressure may adversely affect instrument longevity. Consequently, this study employed a rotation speed of 1200 rpm as optimal.

#### 3.4.2. Impact of Flow Rate of Mobile Phase

**Figure 3:**
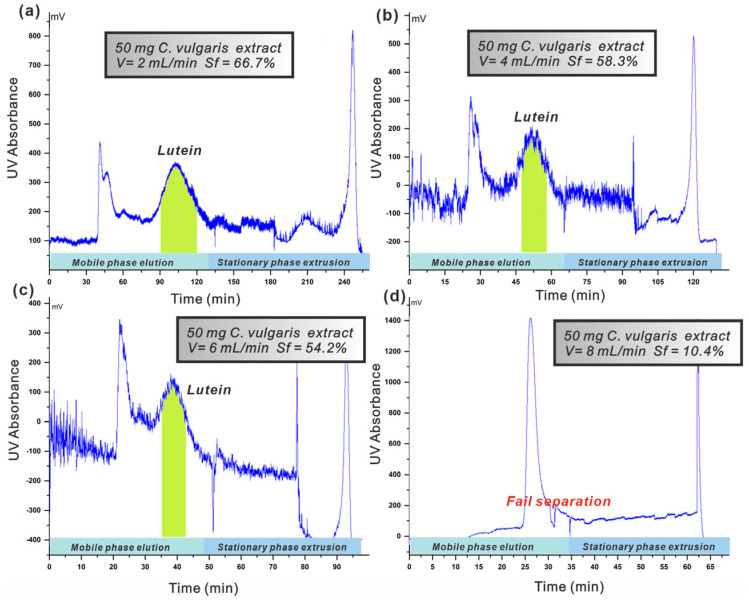
(**a**–**d**): Effect of different mobile phase flow rates on the separation efficiency of lutein from *C. vulgaris* crude extracts (rotation speed: 1200 rpm; liquid system: limonene/ethanol/water 10/9/1 *v*/*v*; stationary phase: limonene-rich organic phase; mobile phase: polar phase in the ascending direction; the green area was the time being collected of lutein).

When the CPC rotation speed is set at 1200 rpm and the injection amount is 50 mg, Figure 3 demonstrates how the flow rate of the mobile phase influences the retention rate of the stationary phase. The figure demonstrates that increasing the mobile phase flow rate generally decreases the stationary phase retention rate. Increasing the flow rate from 2 mL·min^−1^ to 6 mL·min^−1^ results in a gradual decrease in retention rate from 66.7% to 54.2%. For flow rates below 6 mL·min^−1^, this trend continues with a slow reduction in retention value as flow rates increase. At higher flow rates of 6 to 8 mL·min^−1^, the retention rate sharply decreases from 54.2% to 10.4%.

Consequently, it can be concluded that for this system, an optimal upper limit for mobile phase flow rate should be approximately around 6 mL·min^−1^. The mobile phase flow rate should be selected by thoroughly considering factors like stationary phase retention, separation efficiency, and separation time in practical separations. When the CPC rotation speed is set at 1200 rpm and an injection amount of 50 mg is used, Figure 3a–d illustrates the impact of varying mobile phase flow rates on lutein’s separation efficiency. As the flow rate increases, lutein separation decreases. At a flow rate of 8 mL·min^−1^, the stationary phase retention value sharply decreases to 10.4%, complicating the separation of lutein from other impurities. Increasing the mobile phase flow rate shortens the separation time for lutein. Selecting the mobile phase flow rate requires considering factors such as stationary phase retention rates, separation degree, and total separation time. In this experiment, a lutein injection of 50 mg was used, and a final flow rate of 2 mL·min^−1^ was chosen for its optimal separation efficiency and reduced separation time, aligning with the requirements for future large-scale separation.

#### 3.4.3. Effect of the Sample Loading Capacity

**Figure 4:**
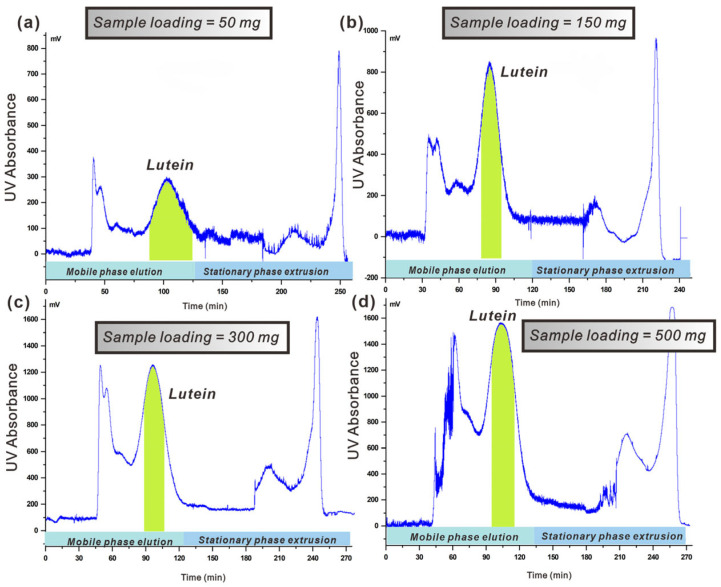
(**a**–**d**): Effect of different loading capacity on the separation efficiency of lutein from *C. vulgaris* crude extracts (rotation speed: 1200 rpm; liquid system: limonene/ethanol/water 10/9/1 *v*/*v*; stationary phase: limonene-rich organic phase; mobile phase: polar phase in the ascending direction; the green area was the time being collected of lutein).

The concentration of the injected sample, referred to as the loading capacity, significantly influences the separation effect. An excessive loading capacity can lead to a loss of stationary phase and reduced resolution, while an insufficient capacity may decrease the yield of target compounds [39]. Therefore, this study investigated the impact of varying loading capacities on separation efficiency. We investigated the impact of varying injection volumes on separation efficiency at a flow rate of 2.0 mL·min^−1^ and a column rotation speed of 1200 rpm. Figure 4 illustrates the findings. The figure demonstrates that increasing the injection amount leads to a rise in lutein’s peak height, while its peak width remains stable, aligning with basic chromatography principles. Concurrently, an increase in injection concentration leads to a gradual loss of stationary phase. As illustrated in Figure 4a–d, at injection amounts of 50 mg and 150 mg, lutein exhibits superior separation efficiency and is effectively separated from other substances. However, with increasing injection amounts, the degree of separation for lutein gradually diminishes. At an injection amount of 300 mg, there is partial overlap between the chromatographic peak for lutein and that for impurities. When increased to 500 mg, this overlap becomes more pronounced, resulting in significantly diminished separation efficiency due to relatively poor resolution between peaks. Consequently, for this experiment, an optimal injection amount was determined to be 300 mg.

### 3.5. Elution–Extrusion CPC Separation of Lutein

#### 3.5.1. Elution–Extrusion CPC Separation

**Figure 5:**
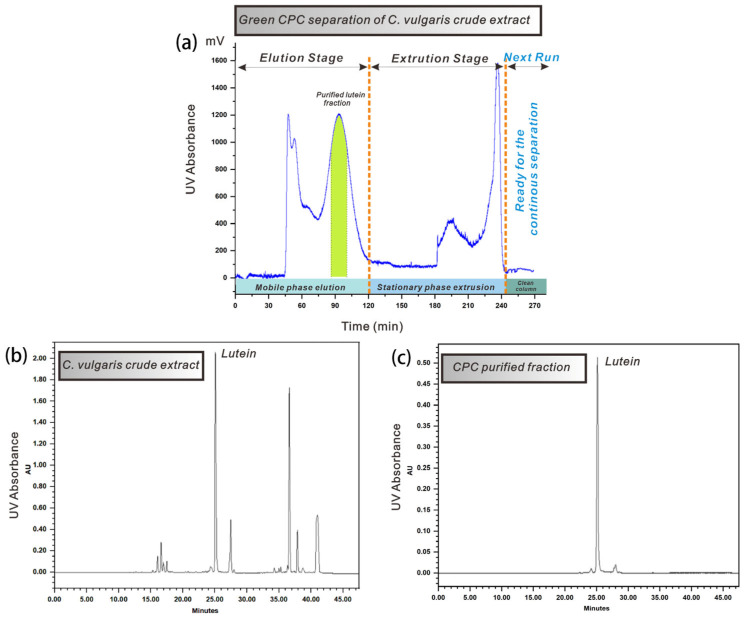
(**a**) Continuous liquid–liquid extraction of lutein from 300 mg crude *C. vulgaris* extract via elution–extrusion CPC using limonene-based biphasic liquid system (rotation speed: 1200 rpm; liquid system: limonene/ethanol/water 10/9/1 *v*/*v*; stationary phase: limonene-rich organic phase; mobile phase: polar phase in the ascending direction; (**b**) the HPLC chromatogram of the crude extract of *C. vulgaris*; (**c**) the HPLC chromatogram of the purified lutein extract (green) using the proposed method.

After the aforementioned optimization, the optimal conditions for purifying lutein from crude *C. vulgaris* extract using a limonene-based green biphasic liquid system have been established. Figure 5 illustrates the CPC separation process for lutein from 300 mg of crude *C. vulgaris* extract utilizing a biphasic solvent system composed of limonene, ethanol, and water in a 10:9:1 volume ratio. The CPC separation was conducted on an instrument with a capacity of 240 mL. A flow rate of 2.0 mL·min^−1^ was used to achieve satisfactory peak resolution, leading to a stationary phase retention (Sf) of 66.0%. As depicted in Figure 5a, a total of 300 mg of crude *C. vulgaris* extract was effectively separated through a single elution–extrusion CPC process, which lasted for a duration of 270 min. Figure 5b,c presents the HPLC chromatograms of both the injected crude *C. vulgaris* extract and the CPC-purified lutein fractions. The lutein content was quantified by analyzing the HPLC peak area using a standard as reference. In the crude *C. vulgaris* extract, the lutein content was approximately 25 mg/g (crude extract). Following CPC separation, the purified lutein fraction (approximately 2.0 mg as indicated by the green portion in Figure 5a) achieved the highest purity level of 94.6%.

#### 3.5.2. Use of Limonene as “Green” Alternative Solvent

Compared to the conventional hydrodynamic CCC columns, hydrostatic CPC columns offer advantages such as enhanced stability and increased sample capacities. This method is particularly well suited for the limonene–ethanol–water solvent system, which demonstrates minimal differences in upper phase density. Consequently, this characteristic significantly improves its effectiveness in the separation and purification of high-purity lutein from microalgae. Experimental results demonstrate that limonene can effectively replace *n*-hexane as the solvent system for CPC in lutein separation. The use of limonene instead of *n*-hexane creates a completely green and natural solvent system that poses no pollution to the environment or harm to experimental operators. Furthermore, the use of limonene as a green solvent has several advantages over traditional solvents, such as chlorinated solvents and hydrocarbons. It is non-toxic, non-carcinogenic, and non-flammable, making it safer and more environmentally friendly. It also has a lower vapor pressure and a higher flash point than many traditional solvents, reducing the risk of fire and explosion. Overall, limonene has promising prospects as a green solvent for various industrial applications. Its unique physicochemical properties and biodegradability make it an attractive alternative to traditional solvents, contributing to the development of sustainable and environmentally friendly processes.

#### 3.5.3. Elution–Extrusion Mode for Continuous and Sustainable Separation

The elution–extrusion protocol, as proposed by Berthod et al. [37], effectively utilize the liquid characteristics of the stationary phase in both CCC and CPC protocols. This method successfully elutes the most retained solutes from the column while maintaining satisfactory peak resolution. Importantly, it significantly reduces solvent consumption and separation time compared to traditional elution methods. Previous research has demonstrated an alternative approach to extrusion: maintaining a consistent mobile phase while back-extruding both the liquid stationary phase and its contained solutes, thereby altering the flow direction of the mobile phase. This technique is referred to as back-extrusion CCC (BECCC) [40]. Both extrusion methods possess the potential to broaden hydrophobicity windows for CCC/CPC techniques, offering a significant advantage over alternative methodologies. This is primarily because band broadening within chromatographic columns is exclusively dependent on band position [41]. The narrow band widths maintained within the column during the extrusion process result in exceptionally sharp peaks. Consequently, the extrusion-based CCC/CPC protocol emerges as an ideal choice for researchers seeking high-resolution separation of complex mixtures. The approach has proven successful in isolating and purifying significant bioactive ingredients from diverse complex natural extracts, including *Evodia rutaecarpa*, *Polygonum cuspidatum*, as well as edible algae *Sargassum fusiforme*, *Laminaria japonica*, and *Undaria pinnatifida* [32,33,40,41].

Furthermore, the elution–extrusion method offers another advantage in that the column is refilled with the original stationary phase after each elution process. This eliminates the need for an additional pumping process of the stationary phase, allowing for continuous injection and separation without interruption. As a result, the CPC column is immediately ready for the next injection, thereby facilitating seamless and efficient operation. This changes the stereotype of CCC/CPC as cumbersome and less automotive, making the possibility of industrial continuous and process separation. Its ability to handle large volumes of sample solutions accelerates production processes, ensures consistent quality and accuracy, and improves overall productivity. Additionally, it contributes to environmental sustainability by minimizing waste generation and maximizing resource utilization.

## 4. Conclusions

In summary, an efficient green method for extracting lutein from *C. vulgaris* has been established. The Craig model CPC apparatus was utilized for continuous liquid–liquid extraction. By employing a limonene-based biphasic liquid system, high-purity lutein (>90% pure) was successfully obtained from crude extract in a single run. The exceptional purity achieved aligns with the stringent requirements for functional food ingredients, particularly in nutraceuticals and dietary supplements targeting vision health and antioxidant enrichment. Furthermore, the elution–extrusion operation mode was employed to fully exploit the liquid nature of the stationary phase in CPC. This proposed protocol offers a sustainable and environmentally friendly alternative for extracting valuable ingredients from microalgae biomass, demonstrating its potential for industrial continuous and process separation applications. The scalability of this method further supports its feasibility in producing lutein-enriched formulations for the growing functional food market. However, the utilization of “non-green” reagents remained during the sample pretreatment stage. In future research, we will systematically promote the optimization of alternative solutions for green solvents across all extraction stages. By screening and evaluating a broader range of environmentally friendly solvent systems, our objective is to enhance the greenness and sustainability of the entire extraction process while ensuring high extraction efficiency.

## Figures and Tables

**Table 1:** Physicochemical properties of the studied solvents.

Parameter	Unit	*n*-Hexane	Limonene	Ethanol
Molecular weight	Dalton	86	136	46
Density	g/cm^3^	0.659	0.841	0.790
Water solubility ^a^	%	0.00024	0.08	miscible
Ethanol solubility ^a^	%	33.3	38	miscible
Boiling point	°C	68.9	178	78.4
Viscosity 25 °C	mPa·s or cP	0.307	0.923	1.096
UV cut-off wavelength	nm	210	250	210
studied biphasic liquid system: oil/ethanol/water (10/9/1, *v*/*v*)				
Density difference ^b^	g/cm^3^	+0.115 (*n*-hexane upper phase)	−0.009 (limonene lower phase)	/
Oil phase	% *v*/*v*	42	43	
Polar phase	% *v*/*v*	58	57	
Ethanol in oil phase	% *v*/*v*	9.9	12.2	
Water in oil phase	% *v*/*v*	0	0.06	
Oil in polar phase	% *v*/*v*	20.7	21.4	
Water in polar phase	% *v*/*v*	8.6	8.8	

^a^ Solvent solubility in water or in ethanol at 25 °C expressed in weight percentage. ^b^ Difference in density between the aqueous phase and the oil (*n*-hexane or limonene) phase of the system oil/ethanol/water 10/9/1 *v*/*v*.

**Table 2:** The partition coefficient (*K_D_*) of lutein in several solvent systems.

Solvent Systems (*v*/*v*)	Partition Coefficient (*K_D_*)
*n*-Hexane–ethanol–water (15:9:1)	0.13
*n*-Hexane–ethanol–water (10:9:1)	0.17
*n*-Hexane–ethanol–water (10:7:3)	1.49
Limonene–ethanol–water (15:9:1)	0.47
Limonene–ethanol–water (10:9:1)	0.75
Limonene–ethanol–water (10:7:3)	10.56

## Data Availability

The original contributions presented in this study are included in the article. Further inquiries can be directed to the corresponding author.
